# Supplementary material for: The RacGAP β-Chimaerin is essential for cerebellar granule cell migration
Source: Sci Rep. 2018 Jan 12;8:680. doi: 10.1038/s41598-017-19116-w (PMC5766509; doi:10.1038/s41598-017-19116-w)
Supplement: Supplementary file 1 — Supplementary information [file 41598_2017_19116_MOESM1_ESM.pdf]

Supplementary Information

The RacGAP βChimaerin is essential for cerebellar granule cell migration

Jason A. Estep[1], Wenny Wong[2], Yiu-Cheung E. Wong[2], Brian M. Loui [2] and Martin M. Riccomagno[1, 2,*]

1. Cell, Molecular and Developmental Biology Program,

2. Neuroscience Program, Department of Molecular, Cell, and Systems Biology, University of California, Riverside, CA 92521, USA.

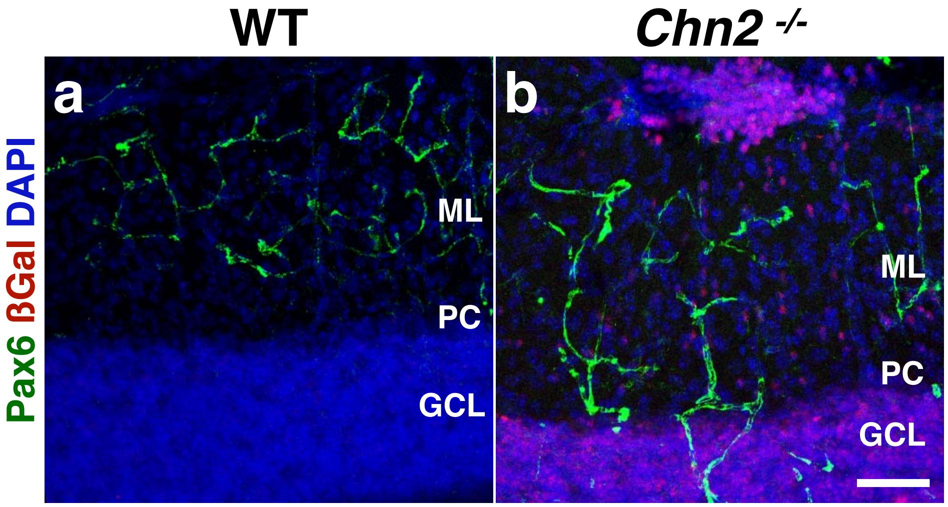

Supplementary Figure S1. Ectopias in Chn2 null animals do not express Pax6. (a, b) Immunostaining of WT (a) and Chn2$^{-/-}$ (b) adult cerebella for Pax6 (green) and βgal (red), with DAPI as counterstain. Ectopias do not display any Pax6 staining. Scale bar, 50μm.
